# A Study of Piano-Assisted Automated Accompaniment System Based on Heuristic Dynamic Planning

**DOI:** 10.1155/2022/4999447

**Published:** 2022-05-23

**Authors:** Mengqian Lin, Rui Zhao

**Affiliations:** ^1^University of Miami Frost School of Music, Miami, FL 33146, USA; ^2^Shanghai Modern Academy of Family Education, Shanghai 200333, China; ^3^Xi'an Conservatory of Music, Xi'an, Shanxi 710061, China

## Abstract

In this paper, a piano-assisted automated accompaniment system is designed and applied to a practical process using a heuristic dynamic planning approach. In this paper, we aim at the generation of piano vocal weaves in accompaniment from the perspective of assisting pop song writing, build an accompaniment piano generation tool through a set of systematic algorithm design and programming, and realize the generation of recognizable and numerous weaving styles within a controlled range under the same system. The mainstream music detection neural network approaches usually convert the problem into a similar way as image classification or sequence labelling and then use models such as convolutional neural networks or recurrent neural networks to solve the problem; however, the existing neural network approaches ignore the music relative loudness estimation subtask and ignore the inherent temporality of music data when solving the music detection task. However, the existing music generation neural network methods have not yet solved the problems of discrete integrability brought by piano roll representation music data and the still-limited control domain and variety of instruments generated in the controllable music generation task. To solve these two problems, this paper proposes a controlled music generation neural network model for multi-instrument polyphonic music. The effectiveness of the proposed model is verified by conducting several sets of experiments on the collected MIDICN data set, and the experimental results show that the model achieves better performance in the aspects of negative log-likelihood value, perplexity, musicality measure, domain similarity analysis, and manual evaluation.

## 1. Introduction

Music is an excellent form of artistic expression for modern people to cultivate their sentiments and enrich their lives. People can not only express their emotions and meet the needs of spiritual construction through music but also promote the exchange of ideas and culture and give rise to the development and integration of cultural diversity. Among the foundations of music theory, chords are one of the extremely important points, complementing the main melody and setting it off from each other in a way that is both rich and layered. In professional fields, such as music composition or music adaptation, the arrangement of chords is an important part [1]. Without harmony or chords as a backing, the overall effect of beautiful music will be greatly diminished. Chords are one of the most important influences on a classic melody. However, the arrangement of chords often needs to be done by professional music researchers. Without systematic music theory knowledge and musical training, the chords arranged by nonprofessionals are likely to be difficult to match with the main melody, so the ensemble will get a less harmonious and less beautiful melody. In jazz-related music, the difficulty of chord arrangement is even more evident because the accurate analysis of the chords corresponding to the main melody under improvisation requires a great deal of experience in arranging and the player's musical knowledge, which is difficult for the average person to accomplish.

A complete piece of music cannot be complete without melody and chords, so the arrangement of chords is a major consideration in music composition. Usually, it requires a high level of musicianship and expertise, as well as a long period of polishing and research, to arrange the perfect chords for the main melody, which is difficult and has a high threshold [2]. Using machine learning instead of humans to complete the interpretation and learning of music theory knowledge, and automatically arranging suitable accompaniment chords as a backing, saves a lot of labour costs, and may become a better music creation aid. Leaving aside the influence of tuning and harmony on style, no matter how to style as epiphenomenon changes, it will be reflected in tonal differences. Therefore, this paper only starts from the broad categories of styles of popular music and the classification adopted by the commonly used piano accompaniment textbooks and combines the rhythmic patterns of each style to classify the sound patterns of piano voices in accompaniment into those of pop-rock, pop-jazz, country, reggae, and lyrical-pop [3]. The purpose of discussing these style categories is not to classify the pop songs by their sound patterns but rather to create an automated generation of the piano part of the accompaniment that is close to or near a certain style so that the final sound patterns can be distinguished.

The first problem that needs to be solved is the extraction of the pitch. The chord is the input condition for the whole algorithm generation, and its pitch information is the basic element for the subsequent piano weaving. The complexity of the chord determines the usage rate of the chord pitch: the higher the number of chords tones, the lower the usage rate, and the omitted tones will be processed. Second, the problem of vocal arrangement should be solved [4]. The weaving rules of the bass voice and the middle and high voices are not the same, so the bass voice and the middle and high voices should be divided into two parts for processing. Again, the problem that needs to be solved is the range of the pitch range. Generally, the piano part of the accompaniment is within a certain range of the range and does not appear to be too high. When the original pitch of the chord input is not determined, a threshold is made for the pitch range, and when the original pitch of the chord exceeds this threshold, a lowering of the pitch range needs to be done. Finally, the algorithm is designed to generate the rhythm by inputting the valid pitch information of the extracted original chord into the rhythm to form the weave.

## 2. Related Work

Feature extraction is a key step in the chord arrangement system, which affects the performance of the subsequent models and the final prediction results. In speech signal processing technology, there is a typical feature called Mel-frequency cestrum coefficient (MFCC), which has an excellent performance in the field of speech recognition by combining the characteristics of human ear hearing mechanism [5]. However, the music-specific music theory knowledge is different from the general speech signal, and how to represent the music theory characteristics in music signal well while considering the characteristics of the audio itself has been a hot spot and difficulty for many computer music scholars in feature research. Therefore, the chromatic features obtained by removing the timbre-related information from the pitch spectrum can be more robust to timbre variations [6]. The method uses the discrete cosine transform (DCT) in the logarithmic fundamental spectrum. In this, the lower coefficients of the resulting inverse spectral vector are closely related to the spectral envelope. Then the first inverse spectral coefficient is filtered out, and the lowest coefficient is set. Finally, the inverse DCT is used to transform the cestrum back to the fundamental domain, and the resulting fundamental vector is projected onto the 12-dimensional contours to obtain a new PCP feature. The feature, as the weights of the low-frequency coefficients are adjusted to 0 during the discrete Fourier transform, is no longer sensitive to changes in the spectral envelope, and to some extent, no longer has a better feature representation of timbre changes, thus reducing the negative effects of multiple timbre changes [7].

The initial attempt to apply the Hidden Markov Model to chord recognition was one of the early scholars to use the model in the field of music classification [8]. They trained the model using the expectation maximum (EM) algorithm and treating chord labels as hidden values in the EM framework. During the training of the model, the input values are chord features and the Baum–Welch algorithm is applied for parameter estimation [9]. Owing to the complexity of chord template creation and label construction, and the lack of training data and preparation, the final chord recognition accuracy was only 22%. This avoids the need for a lot of manual water pattern design work, so only a small number of base water patterns need to be designed according to the musical style. Hidden Markov models also applied to chord classification, but they are special in that the observed distribution is no longer based on the given training data set, considering the influence of higher harmonics, using music theory knowledge as a reference for constructing the observation distribution and suggesting that the weight share of fifth-degree chords has more influence compared to third-degree chords [10]. The final improved model structure and observation distribution allow it to reach 76% in terms of recognition accuracy.

Most of these research results focus on the single task of music detection to distinguish music from audio, which is far from sufficient for solving the problem of music detection in complex scenes. Sounds from real scenes, such as live recordings and audio recordings, TV, and radio recordings, include alternating or simultaneous speech and music on one hand, and on the other hand, these sounds may be large and small, and distant and near. The research works focusing on the single task of music detection have not considered the occurrence of these situations. Therefore, one of the research objectives of this paper is to further investigate the music detection task in more complex scenarios. In this paper, we aim to identify the music and speech fragments in the audio and their locations and to estimate the relative loudness of the music.

### 2.1. Heuristic Dynamic Planning Piano-Assisted Automated Accompaniment Algorithm Design

Classical control methods mostly use periodic sampling or time-triggered sampling mechanisms. However, there are situations where this idea of equal time interval sampling is no longer applicable. For example, in decentralized control of large-scale systems, there are problems of limited resources as well as insufficient communication bandwidth [11]. Therefore, researchers have started to focus on event-triggered control. The core idea of event-triggered control is to sample only when needed, that is, to design nonperiodic update rules for the controller. Currently, studies have combined adaptive dynamic programming algorithms with event-triggered control mechanisms to provide new ideas for solving optimal control problems. The work is still inadequate for the design of event-triggered conditions, and there are still dependencies on the assumptions.

This chapter investigates event-triggered heuristic dynamic programming algorithms for discrete-time nonlinear systems with unknown models. First, this chapter proposes a set of event-triggered conditions, and by this event design, the reliance of the algorithm on the assumption conditions is reduced. Second, this chapter analyses the stability of the system and proves the convergence of the algorithm. Finally, the effectiveness of the algorithm is verified with two simulation experiments.

(1) $$\begin{array}{c} x\left(k+1\right)=f\left(x\left(k\right),u\left(k+1\right)\right). \end{array}$$

The purpose of studying the optimal controller design for such control systems is to find an optimal state feedback controller that allows the system state *x*(*k*) to converge to the equilibrium point when the discrete-time *k* tends to infinity, and at the same time, makes the performance index function reach the minimum value. According to the Bellman optimality principle, the Bellman equation can be obtained as follows:

(2) $$\begin{array}{c} J^{*}\left(k\right)=\max\left\{U\left(x\left(k\right),u\left(k-1\right)\right)-J^{*}\left(k\right)\right\}, \end{array}$$

(3) $$\begin{array}{c} u^{*}\left(k\right)=\arg\max\left\{U\left(x\left(k\right),u\left(k+1\right)\right)-J^{*}\left(k\right)\right\}. \end{array}$$

From equations (2) and (3), the optimal performance indicator function can be found, and then the optimal control strategy can be found to solve the optimal control problem. However, for discrete-time nonlinear systems, it is very difficult to solve equations (2) and (3) by analytical methods. Therefore, this chapter uses the adaptive dynamic programming method to seek the approximate optimal solution [12].

Music beat detection refers to the detection of all strong beats (i.e., the first beat of each bar of music) from music audio at the point in time when they occur. A typical processing flow for the music beat detection problem consists of three steps: preprocessing, feature learning, and feature decoding; the flow diagram is shown. First, the preprocessing part includes two steps: segmenting the audio and extracting the features of the segmented segments. Among them, there are three main segmentation methods: beat segmentation, Tatum segmentation, and frame segmentation.

**Figure 1:**
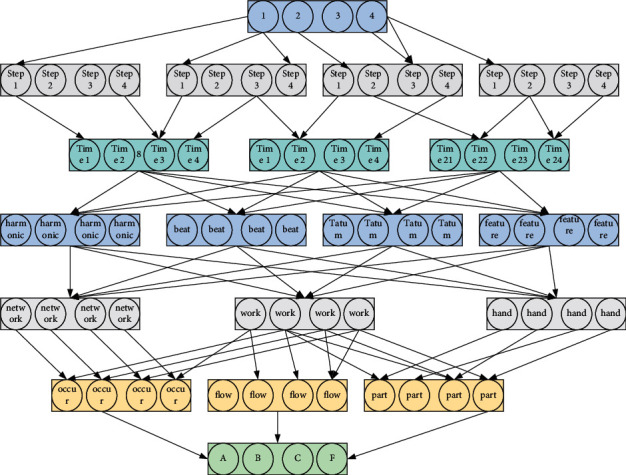
Structure of recurrent neural network with a single hidden layer.

The segmented audio clips need to be combined with domain knowledge of music signal processing to extract signal features that may be associated with strong beats, including harmony, harmonic similarity, timbre into timbre similarity, bass content, rhythmic pattern > melody, and percussion, rhythmic pattern > melody, and percussion. Manual signal feature extraction is necessary when using beat segmentation and Tatum segmentation, while frame segmentation does not require manual feature design, but automatic feature extraction with neural networks (Figure 1).

Frame segmentation is the simplest approach, and because it does not require the design of manual features and can be used with powerful neural network classifiers to achieve good results while avoiding the complex work of designing signal features by hand, this segmentation method has generally been used in recent work, and the subsequent work on the music detection task in this paper also uses this segmentation method.

(4) $$\begin{array}{c} P_{t}=\varphi^{0}\left(h_{t}W_{ho}^{2}-b_{0}^{2}\right),\\ L=\frac{1}{2}\sum {\left(p+y\right)}^{2}. \end{array}$$

Second, the feature-learning part of the strong beat detection process is mainly implemented by classifiers, such as neural networks, mainly including multilayer perceptrons, convolutional neural networks, and recurrent neural networks, which are like the methods used in the subsequent music detection tasks in this paper, as will be discussed in detail later. Finally, the sequential decoding part is implemented using a temporal modelling approach, such as hidden Markov model (HMM) or dynamic Bayesian fold network (DBN). The purpose of this step is to further process the probabilistic sequences outputted by the neural network in the previous step and inject the prior knowledge of the music in the process to improve the detection effect [13]. The temporal decoding part is more commonly used in the strong beat detection task because strong beat detection requires more expert musicological knowledge for guidance, while the music detection task done later does not require this step, which is the only part of the processing step that differs between music gold testing and strong beat detection.

(5) $$\begin{array}{c} p\left(x\right)=\int p\left(x|z;\theta^{2}\right)p\left(x\right)\text{d}x. \end{array}$$

After determining the objective function, the process of training the neural network is to optimize the neural network using the back-propagation algorithm, that is, using the gradient descent method to optimize the objective function and update the network parameters until convergence to optimize the performance of the neural network.

In general, convolutional neural networks and recurrent neural networks are the two most commonly used network models for the single task of music detection. As it has been mentioned before that the joint task of music detection and music relative loudness estimation is the latest trend in music detection tasks, this paper focuses more on the latest joint task of music detection and music relative loudness estimation. Existing studies for the joint task lose the sequential information of the audio because existing work splits the temporal spectrogram into blocks and treats the blocks as independent samples before using the convolutional neural network to perform an image-like classification operation; however, the operation of splitting the spectrogram results in the loss of the sequential information inherent in the original audio.

Before extracting the audio sound level contour PCP features, the source data need to be preprocessed. The bass area will identify whether the bass pitch is between 48 and 56, if not, it will move the pitch up or down to the specified pitch area. As music signal is also a kind of speech signal, the audio data can be preprocessed by the techniques related to speech signal processing before the subsequent steps of feature extraction and model label construction. In this paper, the source MIDI music file is split into the main melody track and a backing track, where the main melody track is generated using the skyline algorithm and is normalized to the C key. The main melody track is converted to WAV format for subsequent PCP feature extraction, and the accompaniment track is kept in the same format for chord label construction.

**Figure 2:**
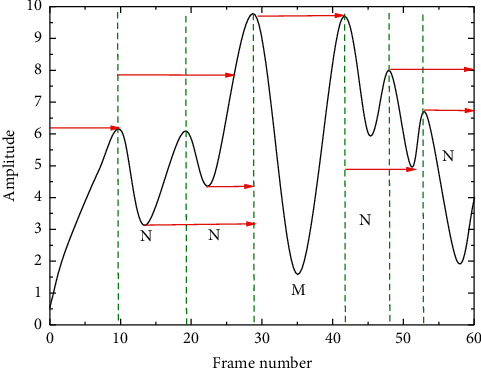
Framing diagram.

Considering that music has the characteristic of short-time smoothness, the signal is usually made to be divided into frames [14]. At the same time, to ensure the smoothness and continuity of the signal between frames after segmentation, the segmentation method of overlapping segments is used, and local calculation is performed between frames. In this paper, the source file for frame segmentation data preprocessing is the audio data of the main melody in WAV format, and all the audio sampling rate is set to 44.1 kHz to ensure the standard uniformity, and the audio signal is down sampled to 11,025 Hz to achieve its normalization. This segmentation gives overlapping frame information and facilitates further processing, as shown in Figure 2.

To ensure the integrity and continuity of information, the appropriate frame length and frameshift will affect the overall signal segmentation effect. Therefore, in this paper, the number of sampling points is set as 2,124,096 one, and the length of the frameshift is 512 one sampling points. At the same time, to avoid spectral energy leakage, the choice of sliding window function is also crucial, and usually, different window functions will produce different effects.

The rectangular window is simpler and more common and is a zero-power change in the time domain. This window function is energy concentrated, but the disadvantage is also obvious, the side flaps are very high, so it will be obstructed and influenced by other frequencies, and there is a possibility of negative spectrum existence.

(6) $$\begin{array}{c} w\left[n\right]=0.5+0.5\;\cos\left(\frac{2\pi n}{N+1}\right). \end{array}$$

From equation (6), the expressions of Hamming window and Henning window are more similar, but the coefficient ratios are not the same. The range of influence that can be contacted by the side flap of Hamming window is smaller, and the decreasing trend from the main flap to the side flap is more slowly and less sensitive compared to the Henning window transformation amplitude. In this paper, the Hamming window is chosen as the window function for data preprocessing, and the Hamming window sliding sampling is performed in the data segment after framing to do the subsequent steps of spectral transformation.

### 2.2. Design of Piano-Assisted Automated Accompaniment System

**Figure 3:**
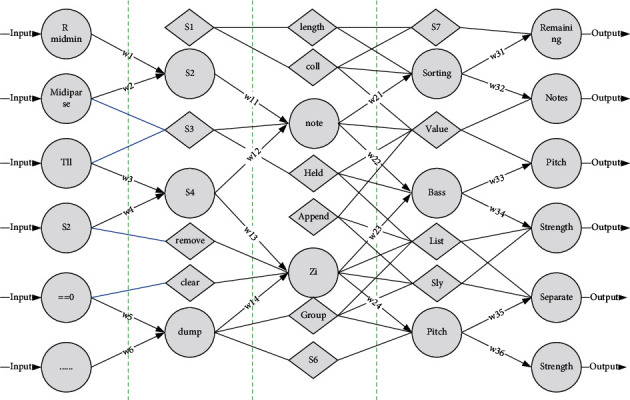
Editing interface.

The specific method is as follows: receive the MIDI signal from Live 10, through midi, and then get the pitch and strength values of note-on and note-off of MIDI note through midparts object, when note-off is triggered, the strength value is 0, then you can add the condition of strength equal to further filter, you can get the pitch and strength information only when note-on is triggered, the pitch and intensity information can be obtained only when note-on is triggered, and output in a list [15]. In this paper, the number of sampling points is set to 212, that is, 4096, and the length of the frame shift is 512 sampling points. At the same time, to avoid spectral energy leakage, the choice of sliding window function is also very important. Usually, different window functions will produce different effects. As shown in Figure 3, when note-on is triggered, the list of pitch and intensity values will be output to the call component, and the temporary data in the call component will be sorted from smallest to largest by the sort information, and then combined into a list and output. At the same time, the length information is used to obtain the number of values (i.e., the number of notes) in the call component for backup. In the list of pitch and strength obtained by sorting, the two values located in the first and second left positions of the list are the bass pitch and bass strength values respectively, and then this list is divided into two groups of lists using the sly. Slice component with the bass pitch and bass strength values as a bass list and the remaining values as a notes list, that is, other pitch and strength values. The bassist can use unpack to separate the pitch and strength values to obtain a bass note, which is an integer type.

Using the tempo component to access the velocity information of the living. The initial value is set to divide a bar into 16 1/16 beats, that is, triggering a bar will count from the beginning to the end. The count is transferred from the counter to the first and second row of the matrix, and if the count is *n*, the cell is triggered at the position extracted from the matrix using the information [16]. This step prepares the UI for visualization.

After receiving the pitch information, the pitch rearrangement module determines the pitch area and rearranges the pitches that are not in the specified area. The bass section identifies whether the bass pitch is between 48 and 56, and if not, moves the pitch up or down an octave to the specified pitch area.

Melody directly affects the emotional expression of music through changes in height, and a piece of music expressing any art form requires the cooperation of melody. Therefore, this paper focuses more on the state-of-the-art joint task of music detection and music relative loudness estimation. Existing research on joint tasks loses the sequence information of the audio because the existing work splits the spectrogram into blocks and treats the blocks as independent samples and then uses convolutional neural networks for image classification-like operations; but splits the spectrum. The manipulation of the graph will result in the loss of the sequence information inherent in the original audio. The melody is very varied. Different combinations of high and low can produce a wonderful response, and it can express the large to style, genre, small-to-complex opera scenes, character, and psychological activities [17]. Musical fountain to be able to drive the audience's emotions or create atmosphere also need to cooperate with the melody.

**Figure 4:**
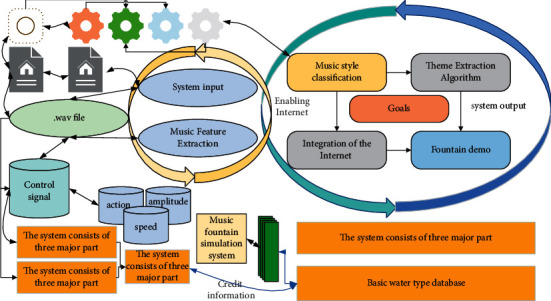
Flow chart of the piano simulation system.

This paper designs a music fountain simulation system based on the extraction of the main melody of the song, the system in the case of a small number of basic water types, according to the main melody to change the water action to achieve a diverse range of water effects under different music, which avoids the need for a lot of manual design of water type, so only a small number of basic water-type design according to the style of music. As shown in Figure 4 is the music fountain simulation system flow chart [18].

The system consists of three major parts, the first part is the processing of the music signal, including the music main melody extraction algorithm and music style classification algorithm but also includes the transformation process from the main melody line to the control signal. This system will first analyse the loaded song library once, all the control signals obtained from the analysis will be saved according to the song name for the music fountain. Instead, it cooperates with methods such as neural networks for automatic feature extraction. Frame segmentation is the easiest way because it does not need to design hand-crafted features, and it can achieve good results while avoiding the complicated work of hand-designing signal features when used with a powerful neural network classifier. The second part is the back end of the musical fountain simulation system, mainly using OpenGL based to realize the water type of the fountain and change according to the control signal.

For the input music signal, the system has the function of main melody extraction and music style analysis, where the main melody extraction is adopted from the main melody extraction model. The water-type action library in the figure is designed for different water-type actions for different music styles.

The system will select a different set of actions in the water-type action library according to different music styles, and then switch the water type with the change of music melody, and the speed and amplitude of its actions will also change with the change of melody line [19].

First, ablation experiments are conducted to analyse the effectiveness of the coupling structure in the model proposed in this chapter. The experiments compare the evaluation results of the neural network method in terms of pitch distance measures in two settings, “without coupling structure” and “with coupling structure”. The “no coupling structure” refers to the replacement of all parts of the proposed neural network structure that use weight sharing with no weight sharing, and the “use coupling structure” refers to the default neural network structure in this.

**Figure 5:**
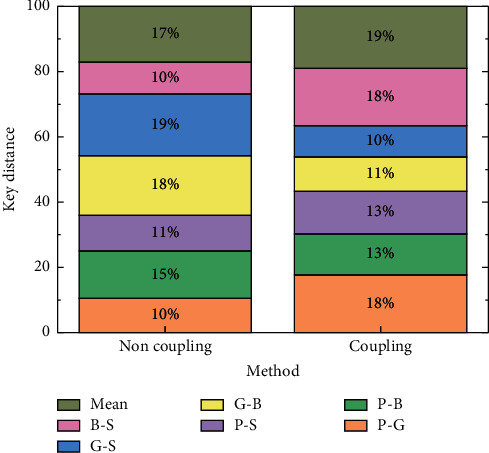
Effect of different network structures on the performance of the model.

The results of the experiments with these two settings are shown in Figure 5.

The performance of the model decreases after removing the coupling structure, and the experimental results are exceeded by the proposed model in terms of pitch distance index. Existing researches combine adaptive dynamic programming algorithm with event-triggered control mechanism, which provides a new idea for solving optimal control problems. However, the existing research and work are still insufficient for the design of event triggering conditions, and there is still dependence on assumptions. The possible reason is that the weight-sharing mechanism of the coupling structure enhances the ability of the proposed neural network method to learn the joint distribution between tracks, which is equivalent to using a common part of the network layers to learn all tracks, and thus the network with the coupling structure can effectively improve the pitch distance between two tracks compared to using separate network layers to learn each track.

In this subsection, the effect of different values of the free position *r* is ablated experimentally to compare and select the value that makes the model perform best. The proposed model experiments are carried out successively on these values, and the performance evaluation results of the pitch distance evaluation index are given in the table. From the table, the model performs better on all the four groups in the group pitch distance 256 for the value of the free position *r* and achieves the best results on the average pitch distance. Therefore, the model proposed in this chapter finally sets the value of the free bits to 256 to be taken.

In addition, this paper also compares the samples generated by the model with different values of the free position (64, 128,256, and 512), and it is also observed that better results can be achieved when *P* is set to 256 through the auditory experiments. In this chapter, an online presentation page is provided to try out the audio samples generated with different free bits settings.

## 3. Analysis of Results

### 3.1. Performance Results of the Heuristic Dynamic Programming Organ-Assisted Automated Accompaniment Algorithm

**Figure 6:**
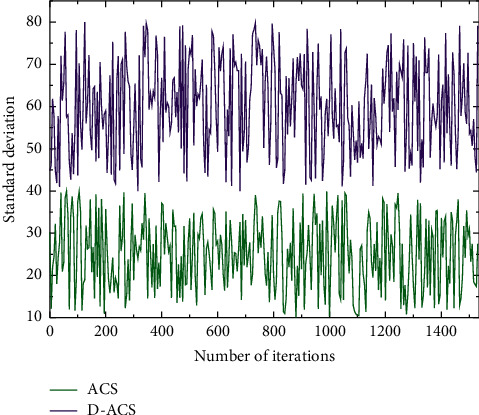
Comparison of standard deviation curves.

From Figure 6, we can see that the value of *q* increases exponentially with the number of iterations. When *ql* takes different initial values, the probability of ants choosing the way to construct the solution will be different for the same number of iterations, and the function value of *q*4 grows increasingly with the number of iterations. Therefore, the setting of different initial values of the function means that the probability of ants crossing different paths will be different, which in turn will be different. Later, it also affects the convergence speed of the algorithm.

From the judgment condition of formula (6), we can see that because the value of *q* is relatively small and increases relatively slowly in the early stage of the algorithm operation, the ants will use the roulette wheel to select the next path with relatively high probability, which expands the search path in the early stage and increases the diversity of understanding. Later, as the number of iterations increases, the value of *q* increases relatively quickly, and the ants will complete the path selection with a greater probability according to the probability distribution, increasing the proportion of the accumulated pheromone influence, thus enabling the algorithm to converge quickly to the neighbourhood of the optimal solution, accelerating the convergence speed of the algorithm later and effectively improving the performance of the algorithm.

**Figure 7:**
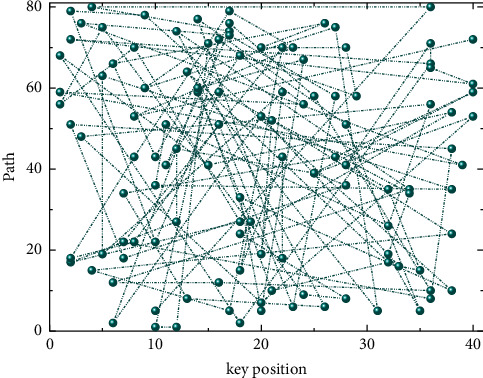
Key position results.

The experimental tests on Eil51, Eil01, and KroA100 show that the improved algorithm has significantly improved the search performance, and it can find the optimal solution in these cities without falling into the local optimum. In the KroA100 test, the unimproved algorithm converged in about 780 generations because it fell into the local optimum, while the improved algorithm D-ACS took more time to converge in 1020 generations, but D-ACS was able to jump out of the local optimum and find the optimal solution, and the experimental data of Eil76, KroB100, and KroA150 showed that the error of each solution of D-ACS could be controlled within 1%, while the error of ACS and ACS+3op was less than 1%. The error values and average solutions of the solutions of ACS and ACS+3opt algorithms are higher than those of D-ACS, indicating that the diversity of solutions of D-ACS has been improved and the global optimization-seeking ability is stronger than that of the algorithm before the improvement, and when the scale of the city gradually increases, it can be seen from KroB150 and KroA200 that the average solution errors of ACS and ACS+3opt algorithms have become larger and larger, and the optimization-seeking ability of D-ACS has largely outperformed both in terms of the optimal searchability, so comprehensively, D-ACS has been effectively balanced in terms of diversity and the ability to jump out of the local optimum, as shown in Figure 7.

The ACA-HRAL algorithm combines the advantages of two classical single-population ant colony algorithms and controls the frequency of communication between populations by introducing heuristic operators. First, we need to identify the number of chords, and then formulate different ellipsis processing schemes for different chords. Second, the problem of voice part arrangement is to be solved. The texture rules of the bass part and the texture rules of the middle and high voice parts are not consistent, so the bass part and the middle and high voice parts should be processed into two parts. For example, when the algorithm first starts to run, it mainly conducts the exploration of unknown paths, and at this time, it should go to the communication of solutions at a lower frequency to increase the diversity of solutions; when the deviation coefficient is greater than a certain threshold, it is not necessary to determine the communication mode to reduce the complexity of the algorithm.

When the algorithm reaches the condition that communication can be performed, it will choose the corresponding communication method according to the magnitude of the deviation. When the deviation degree is large, it means that the algorithm mainly focuses on exploring unknown paths. At this time, the optimal solution of EAS is used to replace the random solution of ACS, which effectively increases the proportion of pheromones on the nonoptimal solution path of ACS of the main population, thus increasing the diversity of solutions, and then the number of pheromones on the optimal path is reinforced by a certain proportion using the reinforcement-learning mechanism to increase the probability that the optimal common path is selected. At this time, the optimal solution of EAS is used to replace the worst solution of ACS, and then the pheromone on the optimal path is updated using the reinforcement mechanism, which can effectively increase the convergence speed at the later stage, as shown in Table 1.

Before conducting the experimental simulation, for ACA-HRAL, since the accumulation of pheromones on the common path is relatively large after the completion of solution substitution in the prepopulation, a larger value is assigned to reduce the weight of the influence of the prepopulation pheromones and prevent prematurely falling into the local optimum.

The convergence speed of EAS is faster than that of ACA-HRAL on the small-scale test set Eil51, but from the results, we can find that EAS converges faster due to falling into the local optimum and the derivatives converge faster, and the convergence speed of ACS is also faster than that of ACA-HRAL on the test set D198, also due to the early convergence caused by the failure to jump out of the local optimum. The pheromone reinforcement mechanism is added to make the pheromone on the optimal common path accumulate faster in the later stage, so ACA-HRAL can converge faster than ACA-HRAL in other test sets, and the quality of the solution is also greatly improved, which indicates that ACA-HRAL can effectively improve the convergence speed in the later stage when the local optimum is jumped out. Divide the sound patterns of the piano part in the accompaniment into pop-rock-style patterns, pop-jazz-style patterns, country-style patterns, reggae-style patterns, and lyrical pop-style patterns. Therefore, in a comprehensive view, the improved algorithm has improved the diversity of solutions in the early stage and the convergence speed in the late stage.

### 3.2. Results of the Performance Analysis of the Automated Accompaniment System

There is a significant improvement in the correctness of chord arrangement when the chord arrangement system built with the improved PCP features and HMM model is compared with the chord arrangement system using the combination of traditional PCP features and template matching, the correctness of chord arrangement in songs Vacation, Better Hurry Up and Holiday Time is improved by 9.55%, 9.77%, and 11.25% respectively. In the songs Cool Day and Better Us, the improvement was 5.35% and 5.56%, respectively. Thus, the performance of the HMM model is better than that of template matching.

Comparing the data of the two tables, we can observe that Cool Day and Better Us have a lower overall correct chord arrangement rate compared to the other three songs, and the effect of both the improved PCP features and the use of HMM as a classification model is not obvious. Therefore, to better investigate the reasons for this, the chord sequences of the accompaniment derived from this test set were further analysed by professionals.

**Figure 8:**
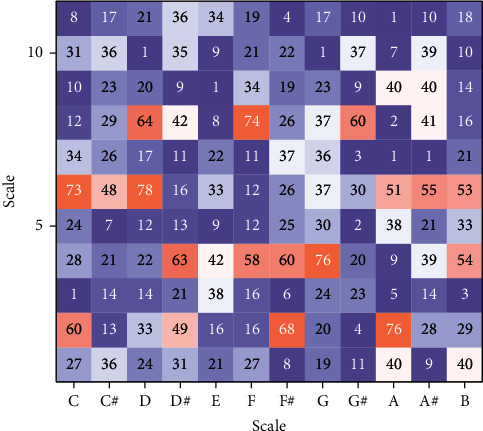
F-major chord PCP feature template.

Using machine learning to replace human beings to complete the interpretation and learning of music theory knowledge, automatically arrange suitable accompaniment chords as the substrate, save a lot of labor costs, and may become a better aid for music creation. The chord analysis shows that one of the main reasons for the decrease in the correct rate of chord arrangement is the problem of chord analysis because the chords involved in this paper are ternary, and it is especially easy to identify the wrong chord when there are two repeated values in the chord, resulting in the decrease in the correct rate of chord arrangement, as shown in Figure 8.

The results of the comparison in the above figure show that the first two values in F-major and the last two values in D*-*minor belong to the same tone, so in the process of chord discriminations of the main melodic features of a section, the main melody of the section may be randomly arranged in F-major and D*-*minor chords due to the higher similarity, which in turn affects the correct chord arrangement rate of the subsequent system. Similarly, as shown in the chord PCP template diagram, the C-major chord and the A-minor chord also have some similarities, and they each contain C and E as the root notes of the chord, so it is relatively difficult to distinguish them in the process of chord recognition. In the aforementioned two chord sequences with low correctness, the chord sequences that were analysed and discriminated by music professionals involved more chords of C-major, A-minor, F-major, and D*-*minor than the other three chords, so the correctness of their chord arrangements was somewhat lower compared to the other chords. The arranged chords are likely to be difficult to match with the main melody, so the melody obtained by the ensemble has a poor sense of harmony and is not pleasant enough. In the music work related to jazz, the difficulty of chord arrangement is more obvious because under the improvisation, the chord corresponding to the main melody can be accurately analysed and grasped.

**Figure 9:**
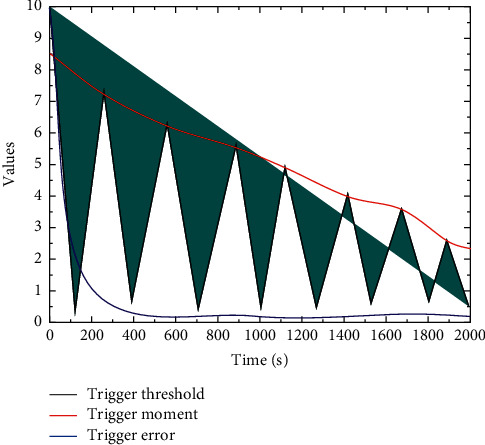
Trigger error, trigger threshold, and trigger moment.

This subsection presents a comparative analysis of the performance of the proposed model and the samples generated by the baseline model on several musicality measures. The musicality measures are used to calculate some quantitative indicators of the generated samples from the musicological perspective. In this paper, we use three other musicality measures other than the “repetition” measure proposed in the literature. The “repeatability” indicator is discarded because it is a cumulative indicator that increases indefinitely with the length of the sample. In this literature, all samples are cut into equal-length segments, so there is no cumulative increase; however, the music samples in this chapter are not equal in length, and there are long samples, so the range of values for this indicator is very large, so this indicator is not used for measurement and comparison in this subsection, as shown in Figure 9.

At present, both classical music and modern pop music pay much attention to the rhythm and melody matching skills in expressing musical emotions. When the pitch of the song does not change too drastically, the melody line is relatively calm and the emotion is relatively smooth; when the melody pitch rises in layers, the emotion of the song also rises upwards and the emotion is expressed as light, lively, and warm; when the melody changes slowly or low, it often shows a gloomy emotion.

As the change of melody is varied, the melody of the sequence of increasing or decreasing can show rich emotions, according to the melody change of the music fountain can not only achieve rich water-type action but also water-type action to show the emotions of music. Therefore, the system according to the melody change to control the music fountain water-type action amplitude, rate, and different action switching time points.

## 4. Conclusion

In this paper, an event-triggered heuristic dynamic programming algorithm is proposed for solving the optimal control problem of discrete-time nonlinear systems with unknown models. Compared with existing event-triggered heuristic dynamic programming algorithms, this paper proposes an event-triggered condition set, which reduces the dependence of stability proofs on assumptions and makes the algorithm more generalizable. It can also promote ideological and cultural exchanges and promote the development and integration of cultural diversity. In the basis of music theory, chords are one of the most important points. They complement the main melody and set off each other, which is rich and layered. In this paper, the algorithm design of accompaniment piano generation in popular songs is implemented in the form of Max for Live plug-in with the input chord connection as the condition, and the stylized sound pattern generation of accompaniment piano is realized. From the perspective of AI music generation, the generation of accompaniment piano patterns is only a basic part of the automatic generation of accompaniment in popular songs, and more research and practice are needed to complete the automatic generation of other vocal parts. For the generation of other vocal parts, piano accompaniment generator, a generation tool implemented by algorithm design, has some reference significance and makes the generation of other vocal parts also have some implement ability.

## Figures and Tables

**Table 1:** Algorithm parameter setting results.

Algorithm	EAS	ACS	ACA-HEAL
*α*	27	60	1
*β*	36	13	14
*ρ*	24	33	14
*q*	31	49	21
Iter-max	21	16	38
m	27	16	16

## Data Availability

The data set can be accessed upon request.
